# Efficient Photocatalytic Degradation of Methylene Blue Dye from Aqueous Solution with Cerium Oxide Nanoparticles and Graphene Oxide-Doped Polyacrylamide

**DOI:** 10.1021/acsomega.3c00198

**Published:** 2023-03-29

**Authors:** Zeynep Kalaycıoğlu, Bengü Özuğur Uysal, Önder Pekcan, F. Bedia Erim

**Affiliations:** † Department of Chemistry, Faculty of Science and Letters, Istanbul Technical University, Maslak, Istanbul 34469, Turkey; ‡ Faculty of Engineering and Natural Sciences, Kadir Has University, Cibali, Fatih, Istanbul 34083, Turkey

## Abstract

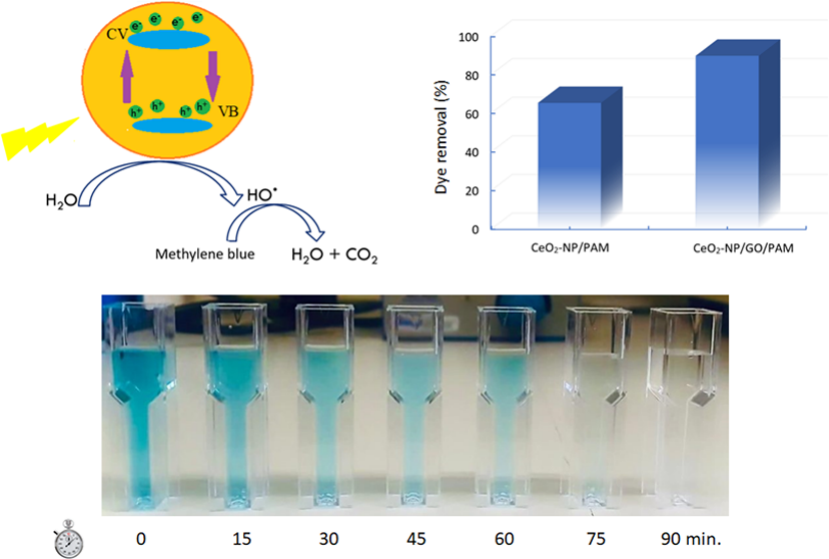

A cerium oxide nanoparticles (CeO_2_-NPs)/graphene oxide (GO)/polyacrylamide (PAM) ternary composite was synthesized through free-radical polymerization of acrylamide in the presence of CeO_2_ nanoparticles and GO in an aqueous system. The synthesized composite material was characterized by X-ray diffraction (XRD), Fourier transform infrared (FTIR) spectroscopy, scanning electron microscopy (SEM), and energy-dispersive X-ray (EDX) spectroscopy techniques and applied for the photocatalytic degradation of methylene blue (MB) dye from an aqueous solution. Tauc’s model for direct transition was used to model for the optical band gap. The key operating parameters such as the amounts of CeO_2_-NPs and GO, pH, initial MB concentration, type of light irradiation, and contact time have been optimized to achieve the highest MB degradation percentage. The photocatalysis process was pH-dependent, and the optimum pH value was found to be 12.0. Under UV-A light, 90% dye degradation occurred in 90 min. The degradation of MB was also specified in terms of total organic carbon (TOC) and chemical oxygen demand (COD). Free-radical capture experiments were also performed to determine the role of radical species during the photocatalytic oxidation process. The photocatalytic process showed that the equilibrium data is in good agreement with the Langmuir–Hinshelwood kinetic model. A rate constant of 0.0259 min^–1^ was obtained. The hydrogel was also tested to assess its reusability, which is an important key factor in practical wastewater treatment. The photocatalytic activity only decreased to 75% after nine uses.

## 1 Introduction

The contamination of water by dyes has been a crucial issue that occupies the world agenda and has an important place in all plans for the future.^1,2^ The increasing amount of these dyes in water causes irreversible harm to aquatic life and indirectly to other living things, organisms, and even humans that depend on them in the life cycle.^3^ The use of reactive dyes has been increasing in various industries. Advanced oxidation technologies (AOTs) have gained importance to remove dyes from water.^4^ The leading one among these techniques is the degradation of dyes by a photochemical process in in situ generating an oxidizing agent in water.^5,6^ Degradation of dyes with the photocatalytic process has several advantages over other methods. The existing conventional methods (i.e., adsorption and chemical coagulation) require further treatment since they convert the dyes from the liquid to the solid phase. Photocatalysis degrades pollutants almost completely, and no secondary pollution occurs. In this regard, the photocatalysis process appears to be more eco-friendly.

Methylene blue (MB) is one of the most widely used industrial dyes; as a result, it is the most common dye in the literature to test the performance of newly developed adsorbent materials. One can effectively eliminate MB from wastewater by utilizing the water-holding capacity, swelling, and adsorption abilities of hydrogels.^7−9^ However, as the hydrogel absorbs water and swells during wastewater treatment, it becomes more elastic, leading to a lower mechanical strength, which is undesirable for the remediation of dye-contaminated water and repeatability. Fortunately, it has proven possible to increase the strength of the hydrogel when incorporated with nanofillers such as metal oxide nanoparticles, nanoclay, carbon nanotubes, and recently graphene.^10−19^ Nanofiller materials with large surface areas not only provide high strength to the new composite when combined with hydrogels^20^ but also enable it to exhibit adsorption abilities on the surface and interior, facilitating the entry of contaminants into the hydrogel. The resulting composite material not only has superior adsorption and dye removal properties but also shows improved properties by forming a stable state and helping regeneration.

CeO_2_-NPs have attracted much attention due to their unique properties. The oxidation state transition between Ce^3+^ and Ce^4+^ found on the surface of CeO_2_-NPs is responsible for the catalytic activity of this nanoparticle. Although the toxicity of nanoparticles is still a questionable issue, it has been shown by experiments that CeO_2_-NPs are not toxic,^21^ and these nanoparticles are compatible with hydrogels and impart durability and antibacterial properties to wound-healing hydrogels and anticorrosive properties to paints.^22−24^ In addition, it has been shown that CeO_2_ nanoparticles synthesized by the green method gain additional and different surface properties.^25,26^ The effective use of CeO_2_-NPs obtained by the green method in dye photodegradation has been lately demonstrated.^26^

In this study, a polyacrylamide (PAM) hydrogel was chosen among the hydrogels because of its synthetic nature with high water uptake capacity and hydrophilicity. PAM forms a soft gel when hydrated. Among the nanofiller materials, cerium oxide nanoparticles (CeO_2_-NPs) and graphene oxide (GO) were chosen in the study with the thought that they would be the best candidates to improve dye affinity and photodegradation.^27^ Since it is difficult to remove metal oxide and graphene oxide from the pollutant medium, the polyacrylamide hydrogel allows to keep them together. Active dye photodegradation of CeO_2_-NPs,^23^ WO_3_/CeO_2_, and Fe_3_O_4_–CeO_2_-mixed NPs^28,29^ has been reported. Lately, a review on the photocatalytic degradation of various pollutants with a CeO_2_ semiconductor as a photocatalyst was reported.^30^ On the other hand, GO is a material with a wide range of applications due to its remarkable properties such as mechanical and thermal strength and electrical conductivity.^31^ Photocatalytic applications of GO also draw attention due to its easily tunable band gap.^31^ It has been reported that GO increases the photocatalytic effect of CeO_2_.^32^ A recent review article demonstrates the photocatalytic applications of GO–CeO_2_ composites.^33^ Stable photocatalyst materials can be obtained by incorporating photocatalytic NPs into hydrogels, eliminating the problem of nanoparticle aggregation in water and particle recovery from water. Moztahida and Lee examined the photocatalytic degradation of MB with a PAM/GO-containing composite and stated that no toxicity from PAM and GO was detected as a result of the toxicity analysis.^34^ Some studies have reported that NPs added into PAM provided dye photodegradation,^34−36^ but we could not find a study of PAM hydrogels in which CeO_2_-NPs were added and used as photocatalysts.

In this study, a ternary polymer composite structure was successfully prepared by first doping CeO_2_-NPs into the PAM hydrogel and then adding graphene oxide (GO), which is an electron transfer material, into the CeO_2_-NP-incorporated PAM hydrogel. The goal of this study is to propose a simple and efficient photocatalytic degradation of MB dye in water with the CeO_2_-NPs/GO/PAM composite hydrogel. The impact of operational factors such as the initial concentration of acrylamide, photocatalyst dose, pH, type of light irradiation, irradiation time, and concentration of dye was evaluated. Comparative experiments were also performed to better understand the combination of GO and CeO_2_-NPs under the same conditions. Consecutive cycle studies were used to assess the reusability of the photocatalyst.

## 2 Experimental Section

### 2.1 Chemicals

N,N′-Methylenebisacrylamide (MBA), *N*,*N*,*N*′,*N*′-tetramethyl ethylenediamine (TEMED), ammonium persulfate (APS), graphene oxide (GO) (2 mg mL^–1^, dispersion in H_2_O), and cerium (IV) oxide (<25 nm) nanoparticles (CeO_2_-NPs) were purchased from Sigma-Aldrich (St. Louis, MO). Methylene blue (MB) and acrylamide (AA) were obtained from Merck (Darmstadt, Germany). Solutions were prepared using deionized water purified using an Elga Purelab Option-7-15 model system (Elga, U.K.).

### 2.2 Preparation of CeO_2_-NPs and GO-Doped PAM Hydrogels

Initially, CeO_2_-NPs (2.5 mg) were added to 5 mL of distilled water and sonicated for 10 min to disperse them. Thereafter, GO (300 μL) was poured into the dispersion and stirred for 1 h to ensure homogeneity. Finally, CeO_2_-NPs/GO/PAM composite hydrogels were prepared by free-radical copolymerization as follows: 500 mg of AA, 10 mg of MBA, 8 mg of APS, and 2 μL of TEMED (0.775 g/mL) were dissolved in the homogeneous suspension of CeO_2_-NPs/GO by stirring at 200 rpm for 15 min. The prepared CeO_2_-NPs/GO/PAM composite was dried at 80 °C. PAM hydrogels were also prepared alone without CeO_2_-NPs and GO for comparison purposes through the same procedure described above.

**Figure 1:**
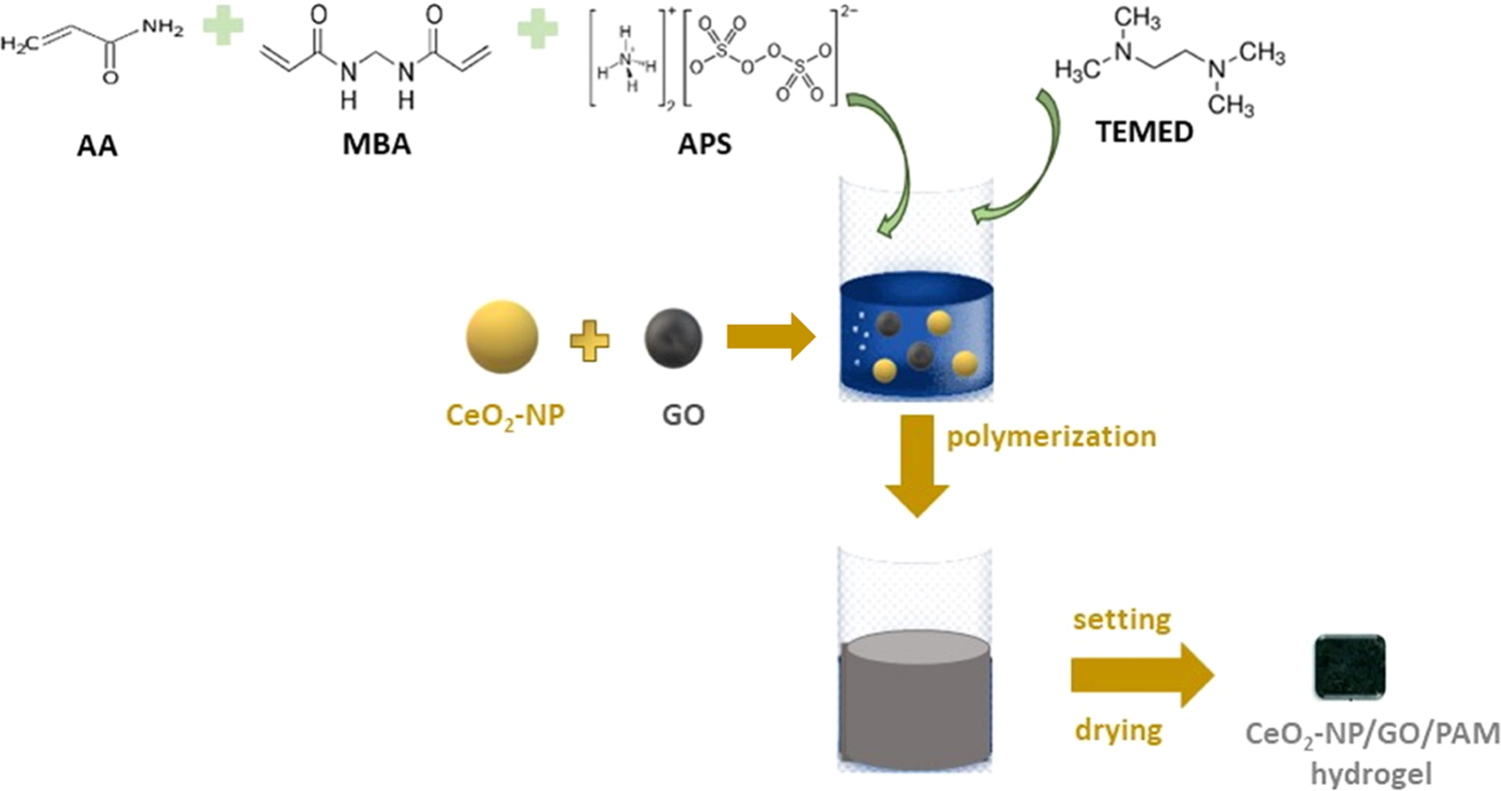
Synthesis diagram of the CeO_2_-NPs/GO/PAM hydrogel.

The synthesis of the CeO_2_-NPs/GO/PAM hydrogel is depicted in Figure 1.

### 2.3 Characterization of the CeO_2_-NPs/GO/PAM Hydrogel

The structural properties of the CeO_2_-NPs/GO/PAM hydrogel were analyzed by Fourier transform infrared (FTIR) spectroscopy using a single reflectance ATR cell by accumulating 64 scans with a resolution of 4.0 cm^–1^. All data were recorded in the spectral range of 4000–400 cm^–1^.

The X-ray diffraction (XRD) pattern of the CeO_2_-NPs/GO/PAM hydrogel was determined using a Rigaku Ultima IV X-ray diffractometer (Tokyo, Japan) using Cu Kα radiation (λ = 1.5418Å, 40 kV, 40 mA).

The particle morphology of the CeO_2_-NPs/GO/PAM hydrogel was investigated using a LEO Supra VP 35 field emission scanning electron microscope (FE-SEM) system, and the elemental composition was estimated by energy-dispersive X-ray (EDX) spectroscopy.

The optical absorbance of PAM, GO-PAM, CeO_2_-NPs/GO/PAM hydrogel solutions, CeO_2_-NPs, and GO was recorded on a Shimadzu 1800 UV–vis spectrophotometer (Tokyo, Japan) in the range of 200–800 nm. In order to obtain the UV–vis spectrum of CeO_2_-NPs, 2.5 mg of the nanoparticles was weighed and dispersed in 5 mL of deionized water using an ultrasonicator for 10 min and magnetic stirrer for 1 h. For the UV–vis spectrum of GO, 300 μL of GO solution was added into the 5 mL of deionized water and dispersed by a magnetic stirrer for 1 h. The band gap energies of synthesized PAM, GO-PAM, and CeO_2_-NPs/GO/PAM samples were determined by the Tauc plots obtained from the absorption spectra according to the following equation
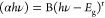
1where α is the absorption coefficient, *h* is the Planck constant, ν is the frequency of the photon, B is the band-tail parameter, and *E*_g_ is the band gap energy.

### 2.4 Photocatalytic Degradation Experiments and Mineralization Studies

For the photocatalytic degradation of MB, 100 mg L^–1^ stock solution of MB dye was prepared in distilled water and mixed until complete dissolution. The solution was stored in a dark place at room temperature. Standard solutions of the dye were diluted from the stock solution. The absorbance measurements were performed with a Shimadzu 1800 UV–vis spectrophotometer (Kyoto, Japan). A calibration curve was constructed by plotting the concentration of standard MB dye versus their absorbance values at 665 nm. The calibration equation obtained from the curve was used to determine the unknown concentration of MB during photocatalytic experiments.

A UV-light cabinet (Kerman Lab, Istanbul, Turkey) with 8 W (UV-A, UV-B, and UV-C) UV lamps was used for photodegradation studies. The lamps were high above 10 cm from the treated MB solution. At regular intervals, the absorbance of a certain amount of MB solution was measured and the taken amount was re-added into the degradation medium. Degradation studies were also performed in sunlight with the same experimental conditions. Furthermore, an experiment was conducted in a dark place in order to understand the adsorption uptake of the hydrogel.

The photocatalytic degradation of MB dye by CeO_2_-NPs and GO was also studied. In order to test the CeO_2_-NPs on MB degradation, 2.5 mg of CeO_2_-NPs was added into 10 mL of 5 mg L^–1^ MB solution. The mixture was ultrasonicated for 10 min and then stirred using a magnetic stirrer for one hour. The photocatalytic experiment was performed by irradiating UV-A light source under vigorous stirring. After that, nanoparticles were separated from the solution by centrifugation at 7000 rpm for five minutes in a Sigma 2–16P centrifuge (Sigma Laborzentrifugen, Germany). For the degradation of MB solution by GO, 300 μL of GO solution was added into 10 mL of 5 mg L^–1^ MB solution, stirred using a magnetic stirrer for one hour, and placed into the UV-light cabinet.

Degradation experiments were done three times. The percentage of MB dye degradation was calculated using the formula below
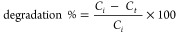
2where *C*_i_ and *C*_t_ are initial and instantaneous concentrations of MB at a certain time, respectively.

In order to obtain the most effective degradation conditions, parameters such as the effect of the pH of MB solution, initial concentration of MB solution, photocatalyst amount, CeO_2_-NP amount, GO amount, and type of light irradiation were investigated. Moreover, the reusability of the CeO_2_-NPs/GO/PAM composite hydrogel was investigated at optimum conditions.

A kinetic experiment for MB dye degradation was carried out under optimized conditions using the absorbance of dye solution measured at 0, 15, 30, 45, 60, 75, and 90 min.

The methylene blue dye was also analyzed for mineralization based on the total organic carbon (TOC) and chemical oxygen demand (COD) removal (%). The TOC analysis was done using a TOC analyzer (TOC-VCPN, Shimadzu, Japan) before and after the photocatalysis of the MB dye using the standard SM 5310B method. The TOC removal efficiency was calculated as
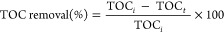
3where TOC*_i_* and TOC*_t_* are the total organic carbon concentrations (mg L^–1^) of the MB dye before and after photocatalysis, respectively.

The COD of the MB dye before and after photocatalysis was analyzed with an MAC COD digester (model: COD-439, Karnal, India) using the ISO 6060 method. The COD removal efficiency was calculated as
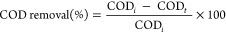
4where COD*_i_* and COD*_t_* are the chemical oxygen demand concentrations (mg L^–1^) of the MB dye before and after photocatalysis, respectively.

### 2.5 Quencher Experiments of the CeO_2_-NPs/GO/PAM Hydrogel

The photocatalysis mechanism was validated by quencher experiments based on the free-radical capture of the CeO_2_-NPs/GO/PAM hydrogel. Silver nitrate, ammonium oxalate, benzoquinone, and *tert*-butyl alcohol were used as e^–^, h^+^, ^•^O_2_^–^, and ^•^OH scavengers, respectively.^37^ The experiments were performed similar to the photocatalytic experiments. The only change in quench experiments was the addition of 1 mmol L^–1^ scavengers to the MB solution.

### 2.6 Determination of the Point of Zero Charge (PZC)

The PZC value is the point at which all active sites become neutral, and so the material’s surface becomes zero. The PZC of the CeO_2_-NPs/GO/PAM hydrogel was determined by the pH drift method. The pH of the 0.1 mol L^–1^ NaNO_3_ was adjusted to a value between 2 and 12 using 0.1 mol L^–1^ HCl or 0.1 mol L^–1^ NaOH.

The hydrogel (0.1 g) was added to 20 mL of the pH-adjusted solution in a capped vial. The vial was shaken up in a shaker and equilibrated for 24 h. The final pH was measured and plotted against the initial pH. The pH at which the curve crosses the pH_initial_ = pH_final_ line was taken as the PZC.

## 3 Results and Discussion

### 3.1 Characterization of the CeO_2_-NPs/GO/PAM Hydrogel

**Figure 2:**
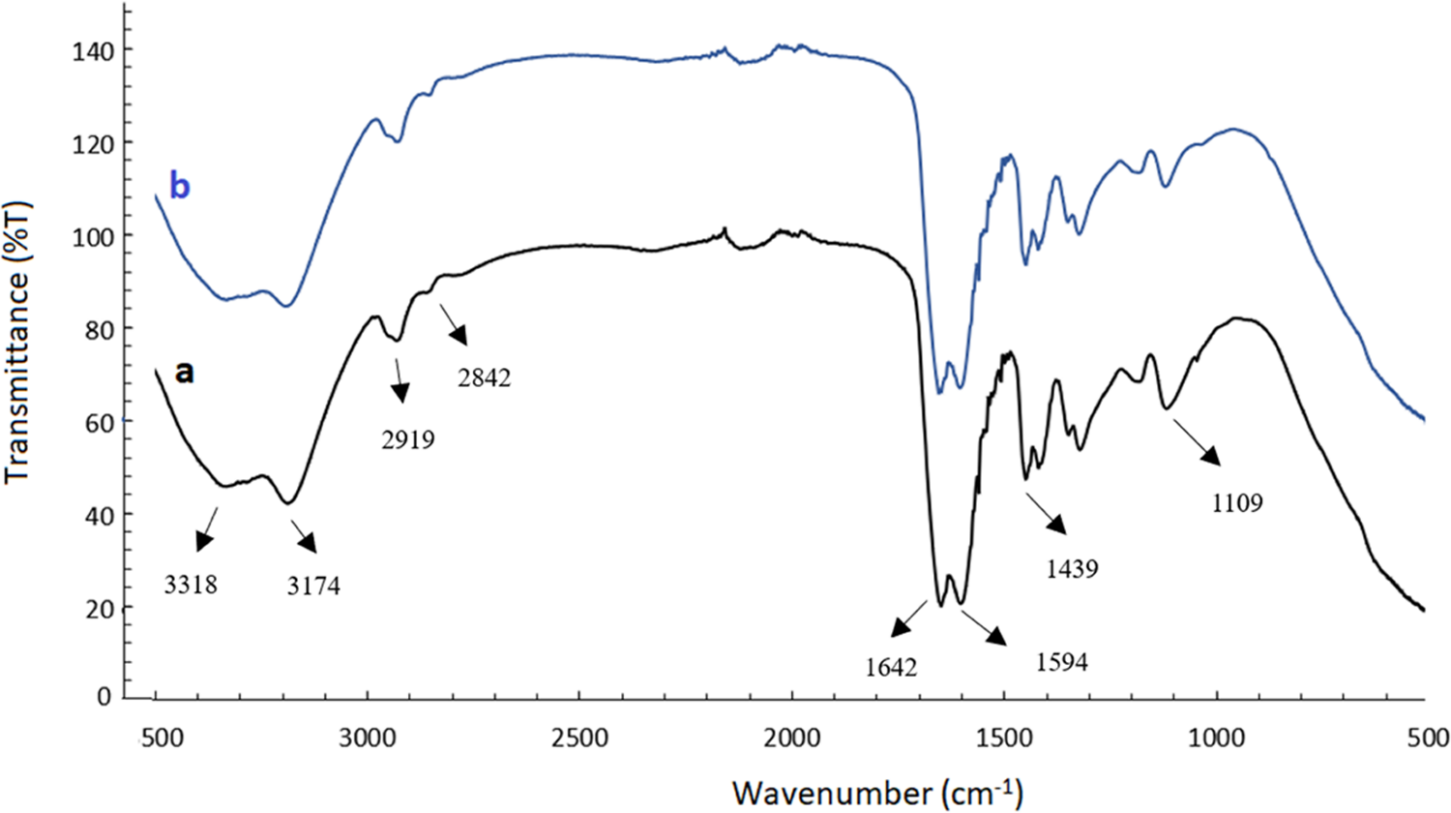
FTIR spectra of the CeO_2_-NPs/GO/PAM hydrogel **(a)** before and **(b)** after photocatalysis of MB dye.

The FTIR spectrum of the CeO_2_-NPs/GO/PAM hydrogel is given in Figure 2a. The main bands occurred at 3318 and 3174 cm^–1^ are attributed to the asymmetric stretching band of NH_2_ and primary amide NH_2_ symmetric stretching band, respectively.^38^ In this region, GO shows a strong and broad O–H stretching vibration band. Secondary amide II overtone was observed at 2919 cm^–1^. The primary amide and secondary amide C=O stretching (CONH_2_) bands occurred at 1642 and 1594 cm^–1^, respectively. The O–H deformation vibration band occurred at 1439 cm^–1^, and the C–O stretching vibration band at 1190 cm^–1^ was observed for GO.^39^ In the case of the spectrum obtained after the photocatalysis of MB (Figure 2b), there is no change of the both intensities and positions of infrared bands. This result confirmed the chemical stability of the hydrogel under photocatalysis.

**Figure 3:**
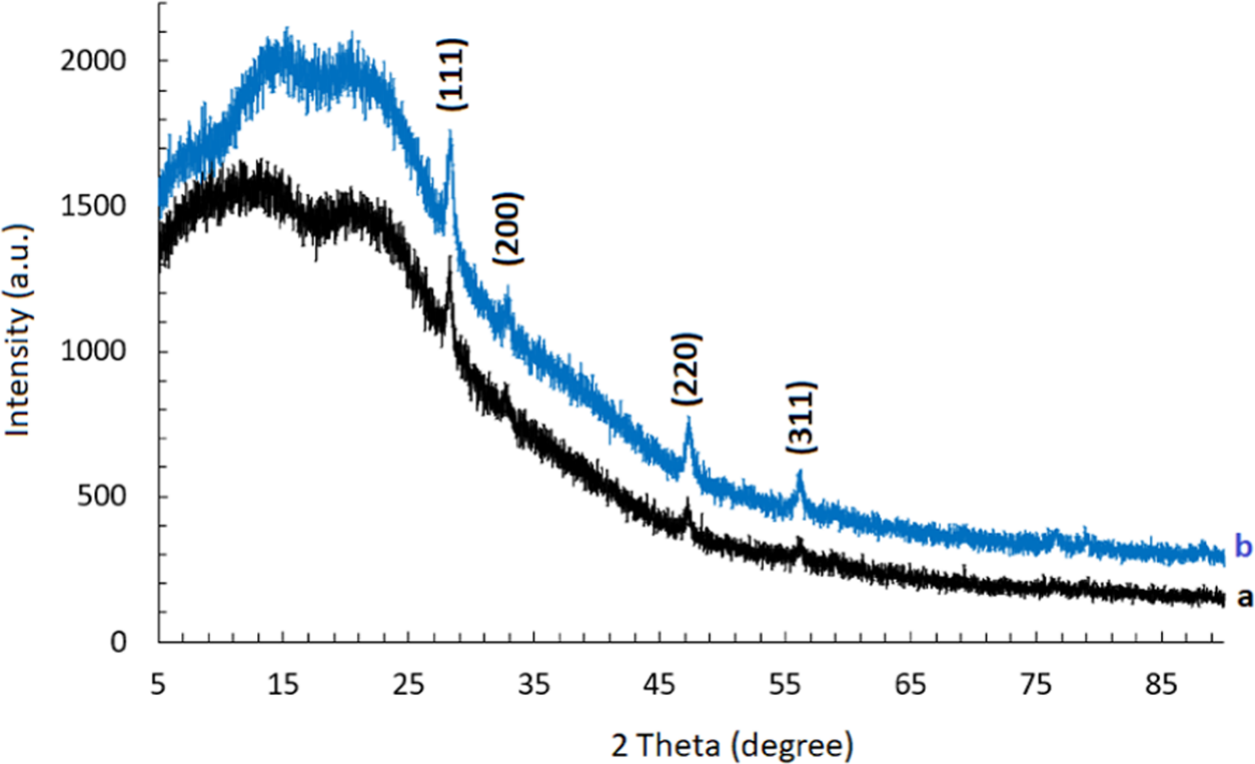
XRD patterns of the CeO_2_-NPs/GO/PAM hydrogels (a) before and (b) after photocatalysis of MB dye.

The X-ray diffraction (XRD) patterns of CeO_2_-NPs/GO/PAM hydrogels before and after photocatalysis are shown in Figure 3a,b, respectively. For the identification of cerium oxide nanoparticles, the peaks were indexed using ICDD No. 34–0394. The diffractograms of the hydrogels are consistent with that of the cerium oxide nanoparticle. All of the distinct peaks at 2 theta (2θ) values of about 28.6, 33.1, 47.5, and 56.3, representing the 111, 200, 220, and 311 Bragg reflections of the face-centered cubic structure of cerium oxide, confirm the presence of CeO_2_ nanoparticles in the hydrogel. These major characteristic peaks confirmed the successful incorporation and uniform distribution of CeO_2_-NPs into the GO/PAM matrix. In addition, no diffraction peaks related to GO were detected, which might be due to its high dispersion in the PAM hydrogel. The XRD profile of the powdered CeO_2_-NPs/GO/PAM hydrogel retrieved after the photocatalytic degradation is similar to that of the powdered hydrogel before photocatalysis. The results of the XRD pattern comparison between before photocatalysis and after the MB photodegradation revealed that the patterns were remarkably similar. No changes in either XRD peak intensities or in the interplanar spacing were observed after the photocatalytic experimentation. This indicates that the CeO_2_-NPs/GO/PAM hydrogel had chemical stability.

**Figure 4:**
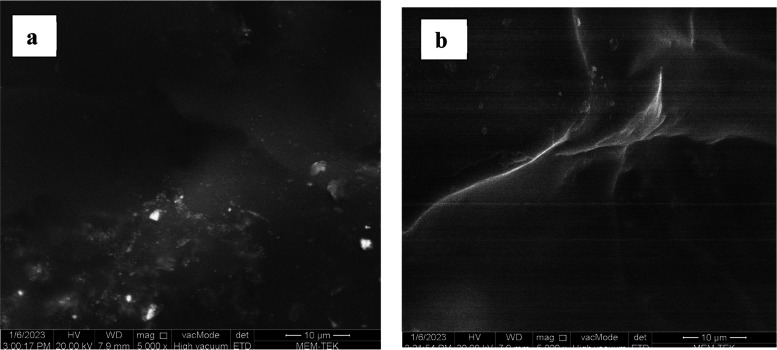
SEM micrographs of the CeO_2_-NPs/GO/PAM hydrogel (a) before and (b) after photocatalysis of MB dye.

The surface morphology of the CeO_2_-NPs/GO/PAM hydrogel was analyzed in SEM. Figure 4a,b shows the SEM micrograph of the hydrogel before and after photocatalysis, respectively. There is no phase separation between polyacrylamide and CeO_2_-NPs/GO, which means that the synthesized hydrogel has a good porous structure. After the photocatalytic process, there is no significant change in the morphological structure of the photocatalyst.

**Figure 5:**
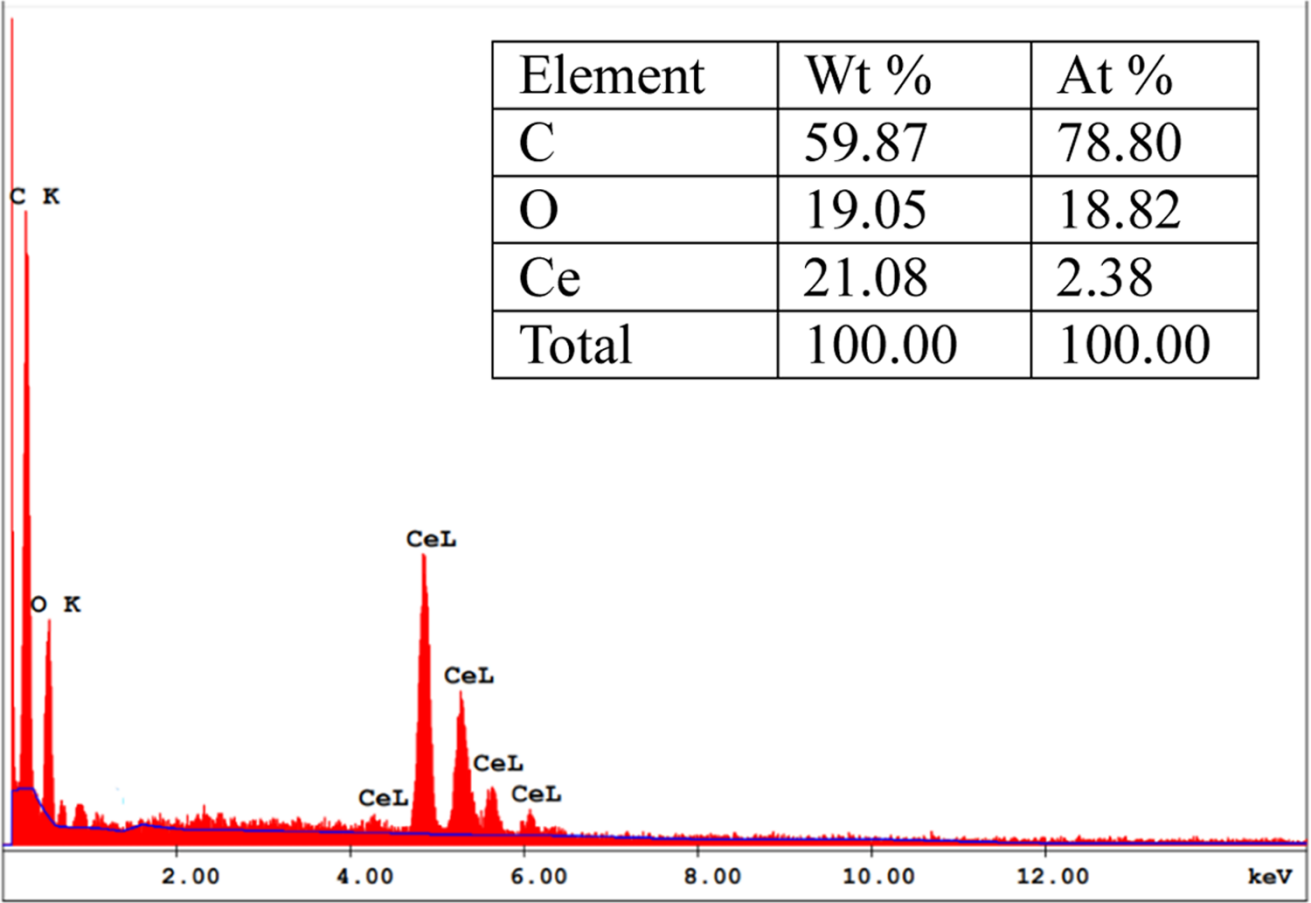
EDX spectra of the CeO_2_-NPs/GO/PAM hydrogel. The inset table shows the EDX quantification.

The energy-dispersive X-ray (EDX) spectrum of the CeO_2_-NPs/GO/PAM hydrogel is shown in Figure 5. The percentages of the elements, i.e., C, O, and Ce are given on the graph.

**Figure 6:**
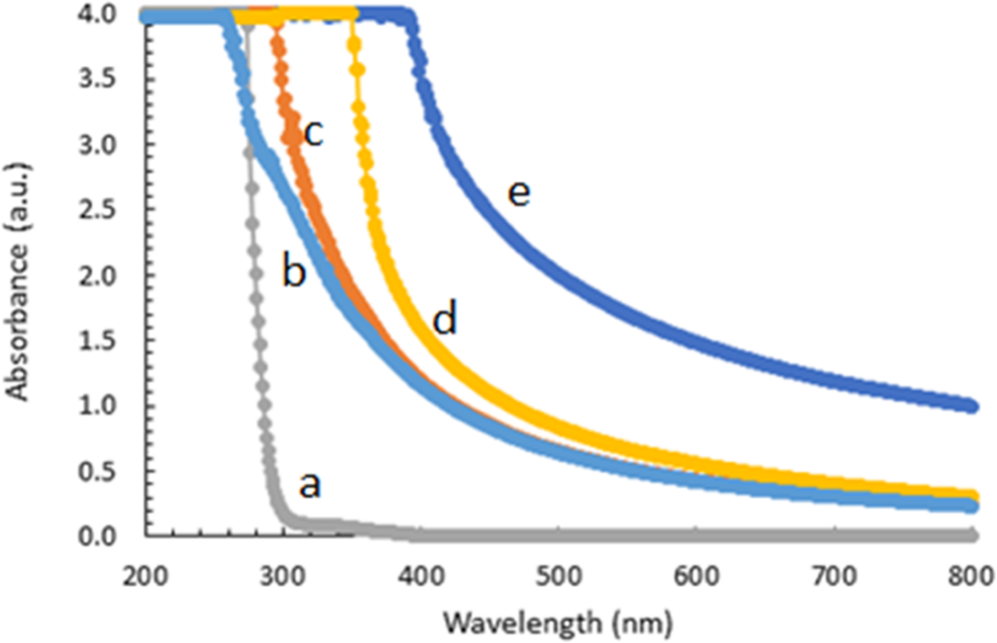
UV–vis spectra of the **(a)**PAM, **(b)**GO, **(c)**GO/PAM, **(d)**CeO_2_-NPs, and **(e)**CeO_2_-NPs/GO/PAM hydrogel solutions.

Figure 6 depicts UV–vis spectra of PAM, GO, GO/PAM, CeO_2_-NPs, and CeO_2_-NPs/GO/PAM hydrogel solutions recorded in the spectrum range of 200–800 nm. The maximum value of absorbance is observed in the UV region, and it decreases with increasing wavelengths for all solutions. In general, when examined in terms of conductivity, PAM is an insulating material. It was tried to obtain a semiconducting material for a photocatalytic activity by inserting CeO_2_-NPs and GO into it.

**Figure 7:**
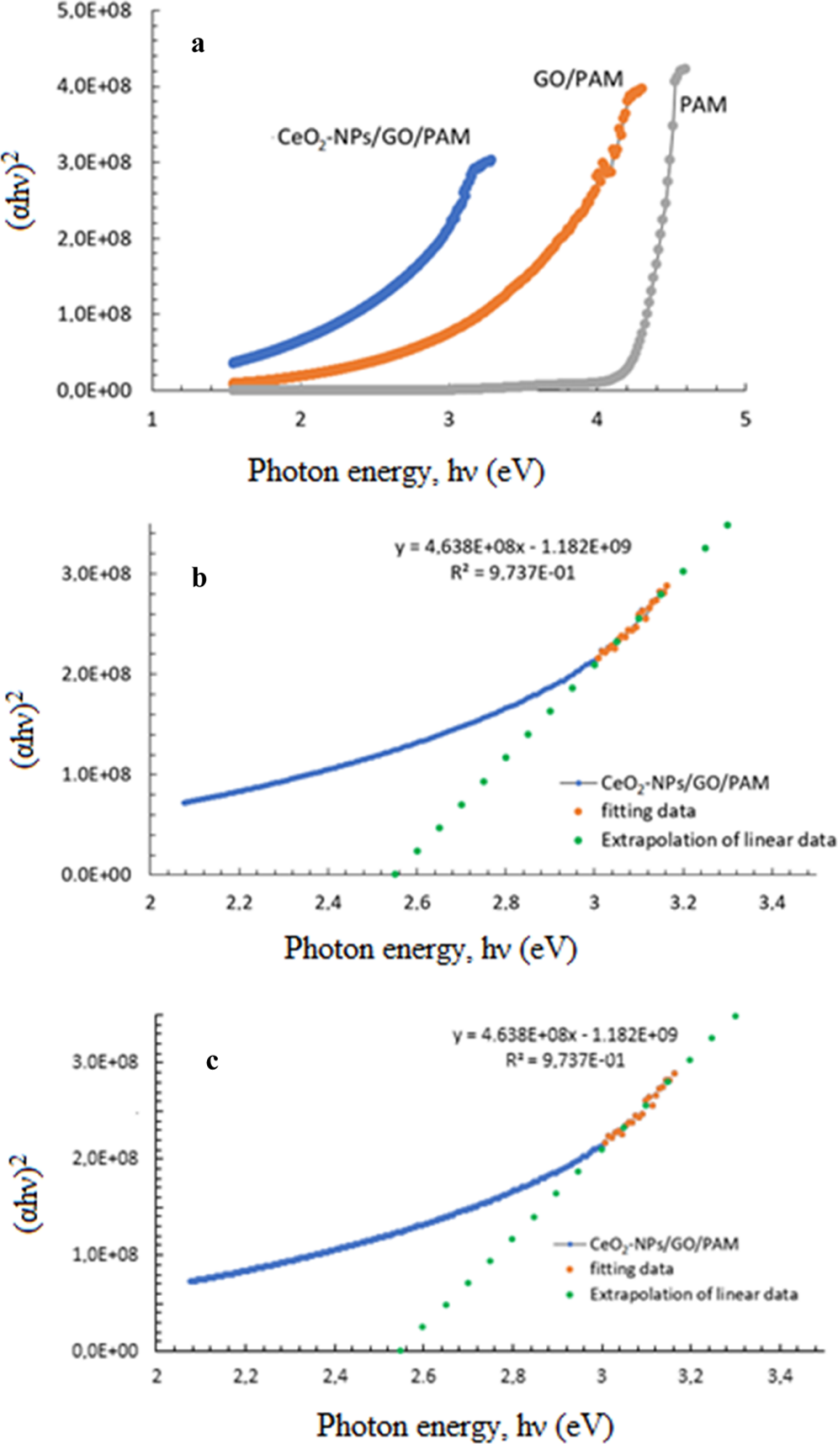
(a) Tauc plots of the hydrogel solutions. Extrapolations of the linear portions of the curves for (b) GO/PAM and (c) CeO_2_-NPs/GO/PAM.

Electronic transitions can occur in direct allowed (r = 1/2), direct forbidden (*r* = 3/2), indirect allowed (*r* = 2), and indirect forbidden (*r* = 3) states. Since CeO_2_-NPs and GO doping inside PAM is considering to manifest a direct allowed transition, in Figure 7, Tauc plots of (α*h*ν)^2^ versus hν are given. In comparison to the absorbance response of PAM, GO addition to PAM presents a red shift in the spectrum due to the narrower band gap energy of GO around 2.2 eV.^40^ For the GO/PAM composite gel, band gap energy is calculated as 3.46 eV from the Tauc plots. CeO_2_-NP addition improves the photocatalytic performance decreasing the band gap energy of the hydrogel to 2.55 eV.

**Figure 8:**
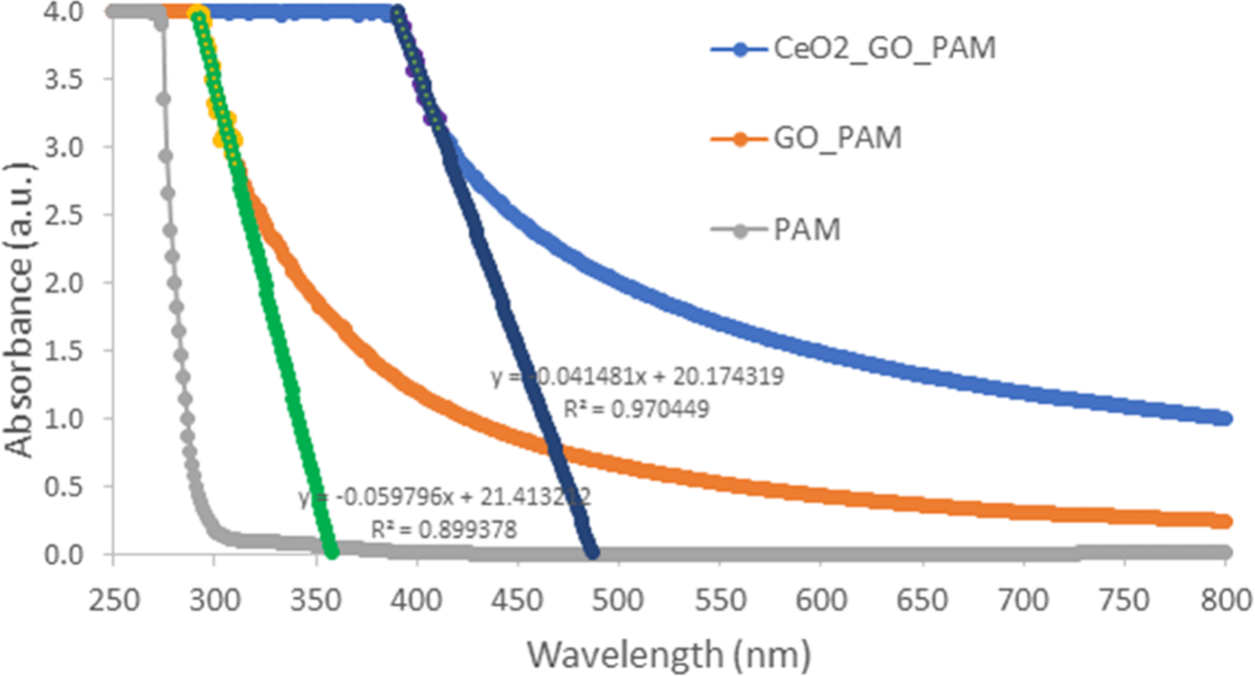
Band gap energy estimation of PAM, GO/PAM, and CeO_2_-NPs/GO/PAM hydrogels.

In addition to the Tauc method, the band gap energy can be calculated approximately from the absorbance–wavelength curve. The absorbance–wavelength curve usually gives a peak if the material contains quantum dots (or particles with about 10–20 nm). Band gap energy can be calculated according to the hc/λ formula of the wavelength equivalent of the peak (maximum of absorbance) corresponding value on the x-axis.^41^ In composite materials containing nanoparticles, especially polymer composites, the absorbance curve sometimes does not have a peak. In this case, for the calculation of band gap energy, extrapolation is made from the region where the curve first flattens to the wavelength axis, and approximately the band gap energy value of the composite is calculated according to the hc/λ formula of the wavelength at which it intersects. In this case, as a result of the extrapolations done in Figure 8, the wavelength values for GO/PAM and CeO_2_-NPs/GO/PAM were found to be 358.1044 and 487.2894 nm, respectively, and the corresponding band gap energy values were 3.46 and 2.44 eV. These values were found to be compatible with the band gap values calculated from the Tauc plots (Figure 7). Therefore, it can be verified that the electronic transition behavior is directly allowed transition.

### 3.2 Effect of the Amounts of CeO_2_-NPs and GO on Photocatalytic Degradation

**Figure 9:**
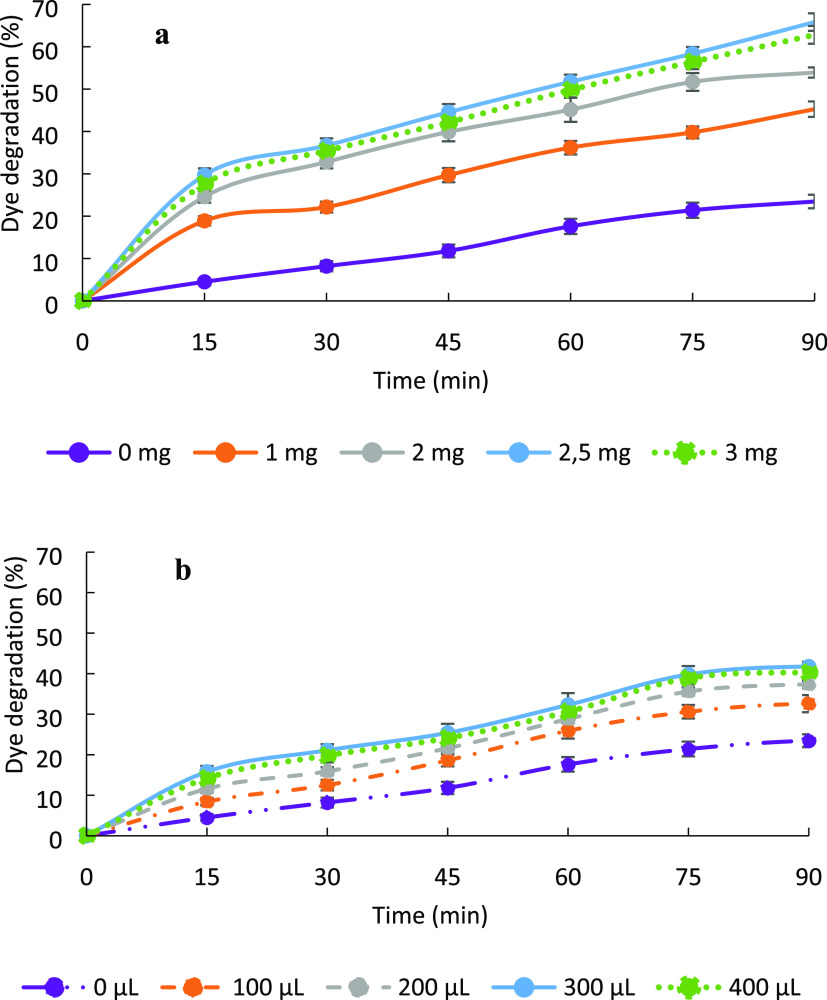
Effect of the amount of (a) CeO_2_-NPs and (b) GO in the PAM hydrogel on the photocatalytic degradation of MB dye.

The effect of the amounts of CeO_2_-NPs and GO on the photocatalytic degradation of MB dye is given in Figure 9a,b, respectively. The dye removal in PAM-added solutions appears to be about 20% under UV light. When the experiment was repeated by exposing methylene blue solution to UV light without the addition of catalysis, degradation at this rate was also detected. Therefore, while PAM has no effect on degradation, CeO_2_-NP addition to the PAM hydrogel from 1 to 2.5 mg, significantly increased the degradation percentage of MB. However, with 3 mg of CeO_2_-NPs, a slight decrease was seen in the degradation percentage. Thus, the optimum CeO_2_-NP amount was determined as 2.5 mg. The PAM hydrogel doped with 2.5 mg CeO_2_-NPs degraded MB dye as 65%.

Subsequently, the effect of GO addition to the hydrogel system on the degradation rate was studied with 100–400 μL of GO solution. The MB degradation increased when the GO amount increased from 100 to 300 μL. However, a slight decrease was observed with the addition of 400 μL of GO. The GO amount was adjusted to 300 μL. Therefore, the photocatalyst was prepared using the following: 500 mg of AA, 10 mg of MBA, 8 mg of APS, 2 μL of TEMED (0.775 g mL^–1^), 2.5 mg of CeO_2_-NPs, and 300 μL of GO. All photocatalysis studies were carried out with this composition.

To find the optimum CeO_2_-NPs-to-GO ratio on photocatalytic activity, the ratios ranging from 1:30 to 1:400 (CeO_2_-NPs-to-GO and weight-to-volume ratios, mg μL^–1^) were studied. The optimum CeO_2_-NPs-to-GO ratio was found to be as 1:120 (w/v). Thus, 2.5 mg of CeO_2_-NPs were mixed with 300 μL of GO, and after that, the hydrogel synthesis was performed.

### 3.3 Effect of pH and the Initial Concentration of MB Solution

The effect of pH on the photocatalytic activity of composite hydrogels was investigated between pH 2.0 and 12.0 using 5 mg L^–1^ MB dye as the initial dye concentration and 0.3 g of the dried hydrogel as the photocatalyst amount. The pH of 5 mg L^–1^ MB dye was measured as 6.0, and this solution was adjusted to the mentioned pH values using 1 mol L^–1^ NaOH or 1 mol L^–1^ HCl. For the experiment performed at pH 6.0, pH adjustment was not performed.

**Figure 10:**
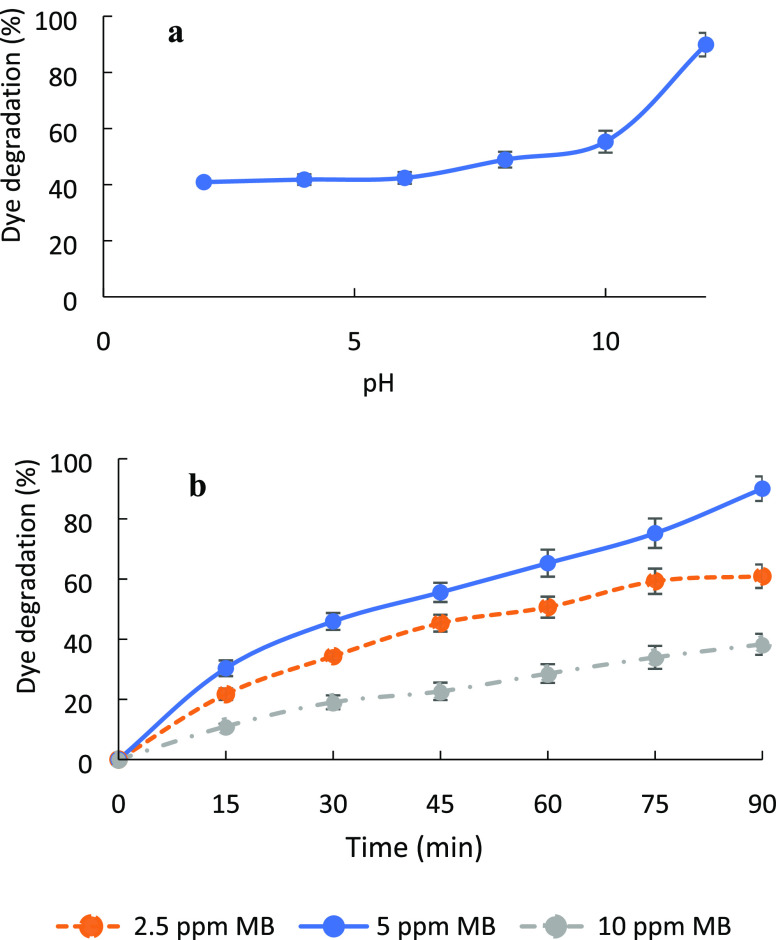
Effect of (a) pH and (b) initial concentration of MB solution.

Figure 10a shows % degradation vs. time for varying pH values of 5 mg L^–1^ MB solutions under UV-A light. The highest degradation percent was obtained at pH 12 in 90 min. It is known that the surface charge of CeO_2_-NPs is sensitive to pH, and at pH 12, the surface charge of particles is negative.^22^ Since the point of zero charge determined by the pH drift method of the CeO_2_-NPs/GO/PAM composite was pH 3.0, the surface charge in the composite is negative. MB is a cationic dye, so a strong electrostatic interaction between the dye and the nanoparticles occurs. This interaction further initiates the photocatalysis effect of CeO_2_-NPs.

The effect of the initial dye concentration on the photocatalysis of MB was tested in the concentration range of 2.5–10 mg L^–1^, keeping the photocatalyst amount at 0.3 g and MB solution pH at 12. When the initial concentration of MB solution increased from 2.5 to 5 mg L^–1^, the percentage of dye removal increased as shown in Figure 10b. However, when the methylene blue concentration increased to 10 mg L^–1^, a decrease in photocatalytic activity was observed. This could be due to methylene blue molecules obstructing the portions of the cerium oxide surface that are triggered by light. Therefore, the formation of ^•^OH radicals, which cause the dye to be oxidized and thus degraded, may be prevented. Thus, the optimum MB concentration was selected as 5 mg L^–1^.

### 3.4 Effect of the Type of Light Irradiation

**Figure 11:**
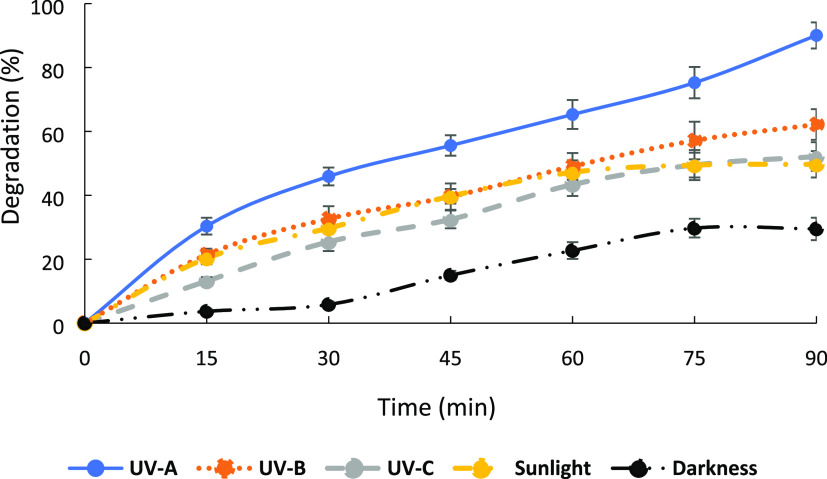
Effect of the type of light irradiation on MB dye degradation.

The effect of the type of light irradiation on MB dye degradation is shown in Figure 11. Under UV-A light, 90% of dye degradation occurred in 90 min, while 50% of the dye degraded in 90 min under sunlight. This result shows that sunlight had no significant effect on MB dye degradation.

For further experiments, the effect of different UV-light sources was investigated. Photocatalysts containing MB dye solutions were irradiated by UV-A (315–400 nm), UV-B (280–315 nm), and UV-C (100–280 nm) light sources. As shown in Figure 11, the maximum dye degradation (90%) was obtained under UV-A light irradiation with the shortest equilibrium time (90 min).

The experiment was also conducted under dark conditions in order to understand the adsorption process between the positively charged MB dye and neutral CeO_2_-NPs/GO/PAM hydrogels. Only ∼30% of dye was degraded with adsorption in 90 min.

### 3.5 Photocatalytic Degradation Mechanism of MB Dye by CeO_2_-NPs/GO/PAM

According to the common pathway, the photoexcitation of CeO_2_ in an aqueous solution during the photocatalytic degradation of contaminants leads to the formation of different radicals and charged species.^30^ The proposed mechanism of dye degradation involves a series of reactions as given in eqs 5–8.

When photons are absorbed by CeO_2_ electrons, hole pairs can be generated inside CeO_2_.
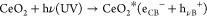
5Here, CeO_2_* is the excited state of CeO_2_, e_CB_^–^ is a photoexcited electron in the conduction band, and h_VB_^+^ is a photogenerated hole in the valence band.^30^

Photogenerated holes directly oxidize the dye to reactive intermediates as shown in eq 6. In the indirect process, the oxygen and water molecules are adsorbed on the photocatalyst’s surface. These molecules react with the electron–hole pairs (eqs 7 and 8) to produce the unstable hydroxyl radicals (^•^OH) and superoxide ions (^•^O_2_^–^), which oxidize the organic pollutants into the inorganic compounds (eqs 9 and 10).
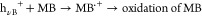
6or
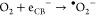
7
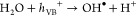
8and
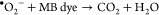
9
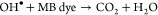
10

Moreover, GO works as an electron acceptor, which helps in the transfer of photoexcited electrons from the CeO_2_ conduction band to GO.^32^ This could lead a synergetic effect on the photocatalytic degradation of MB.

**Figure 12:**
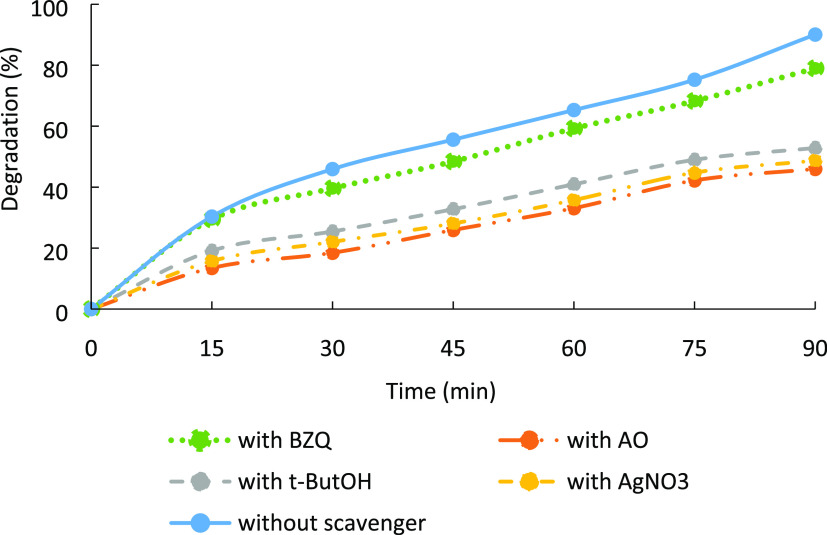
Photocatalytic degradation of MB dye under different scavengers.

In order to further verify the methylene blue degradation and identify the photocatalytic degradation pathways, free-radical capture studies were performed by using silver nitrate (AgNO_3_), ammonium oxalate (AO), benzoquinone (BZQ), and *tert*-butyl alcohol (*t*-ButOH) as e^–^, h^+^, ^•^O_2_^–^, and ^•^OH scavengers, respectively. The results are given in Figure 12. As shown in Figure 12, when 1 mmol L^–1^ BZQ was added into the 5 mg L^–1^ MB dye, the hydrogel degraded the dye as 79%. This value is close to the obtained result from the media without any scavenger (90%). It means that ^•^O_2_^–^ is the main reactive species in the photocatalytic degradation of MB dye by the CeO_2_-NPs/GO/PAM hydrogel. Moreover, a significant inhibition of MB photocatalytic degradation was also observed in the presence of *t*-ButOH, which shows the role of ^•^OH in the photocatalytic process. The MB was degraded as 53% by the introduction of 1 mmol L^–1^*t*-ButOH to the photocatalysis media. The effects of the pathway with the participation of e^–^ and h^+^ were more or less the same. The results indicated that the degradation efficiency decreased to 49 and 46%, with the addition of AgNO_3_ and AO, respectively.

Since CeO_2_ is characterized by a relatively high band gap energy and high rate of e^–^/h^+^ pair recombination, it has been widely applied in the photocatalytic degradation of MB dye. Examples of CeO_2_-NP literature application as single photocatalysis in MB degradation are presented in Table 1. Only one study showed a degradation efficiency of 95% using a CeO_2_-NPs/GO composite as the photocatalyst but longer time compared to our result.^27^

**Table 1:** Degradation of MB by CeO_2_-NPs Obtained from Previous Studies

photocatalyst	degradation efficiency (%)	reaction time (min)	initial MB concentration (mg L^–1^)	ref
CeO_2_-NPs	83.9–93.4	105	5	(23)
CeO_2_-NPs	95	125	5	(42)
CeO_2_-NPs	90.4	90	20	(43)
CeO_2_-NPs	77	210	12.5	(44)
CeO_2_-NPs	80	120	10	(45)
CeO_2_-NPs/GO	95	120	20	(27)
CeO_2_-NPs/GO	90	90	5	this study

**Figure 13:**
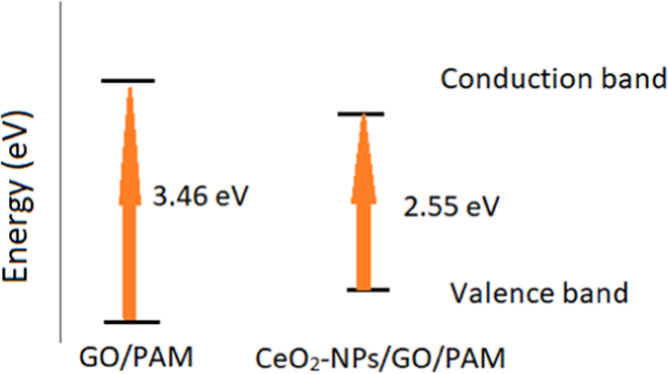
Band gap diagram of GO/PAM and CeO_2_-NPs/GO/PAM constructed from UV–vis spectroscopy and Tauc plots.

Expressing the band gap shift as a function of photocatalytic degradation ability is also insightful. Thus, Figure 13 gives the band diagrams of the hydrogels with band energies obtained from Tauc plots.

### 3.6 Methylene Blue (MB) Mineralization

The amount of MB mineralization is measured by the reduction in total organic carbon (TOC) and chemical oxygen demand (COD). For this purpose, TOC and COD tests were performed to determine the mineralization of the MB dye using the CeO_2_-NPs/GO/PAM hydrogel.

A remarkable decrease in TOC was obtained that implies efficient mineralization of MB with CeO_2_-NPs/GO/PAM. The TOC decreased from 70 to 6 mg L^–1^, which corresponded to 91% removal of the MB dye. This result was supported by experimental photocatalytic degradation of the MB (90%). Similarly, the decrease in COD reveals the degree of associated organic species’ mineralization. The degradation of the MB dye was 78% when the COD decreased from 220 to 47 mg L^–1^.

### 3.7 Kinetic Modeling of Dye Degradation

The Langmuir–Hinshelwood kinetic model was applied to experimental data.^5,46^ This model covers the kinetics of heterogeneous catalytic processes. The degradation experiments by UV-A irradiation of MB solutions containing CeO_2_-NPs/GO/PAM composite hydrogels follow the pseudo-first-order kinetics
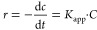
where *r* is the photocatalytic degradation rate (mg L^–1^ min^–1^), *C* is the concentration of the dye in the bulk solution (mg L^–1^), *t* is the irradiation time, and *K*_app_ is the apparent degradation rate constant (min^–1^). Integration of this equation gives the following relation
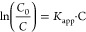
in which *C*_0_ is the initial concentration in the bulk solution.

**Figure 14:**
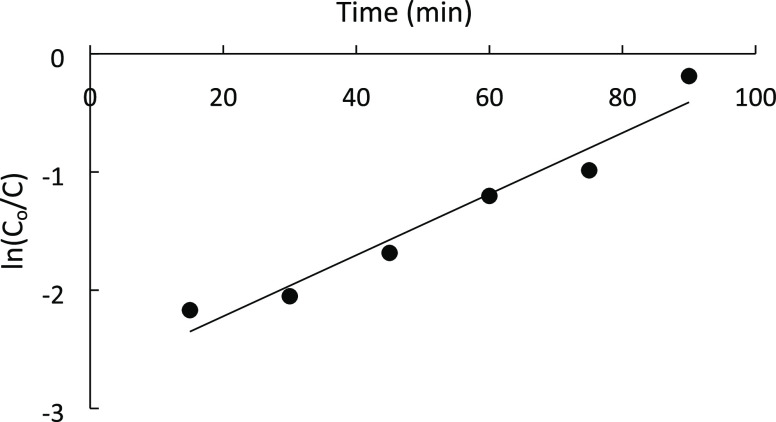
Langmuir–Hinshelwood plot for photodegradation of MB with CeO_2_-NPs/GO/PAM.

It is found that the ln(*C_t_*/*C*_0_) vs irradiation time plot follows a linear kinetic relationship as shown in Figure 14. The reaction rate constant of degradation as the slope of the line was 0.0259 min^–1^. The regression coefficient (*R*^2^) was found to be 0.9504.

### 3.8 Reusability of CeO_2_-NPs/GO/PAM Hydrogels

**Figure 15:**
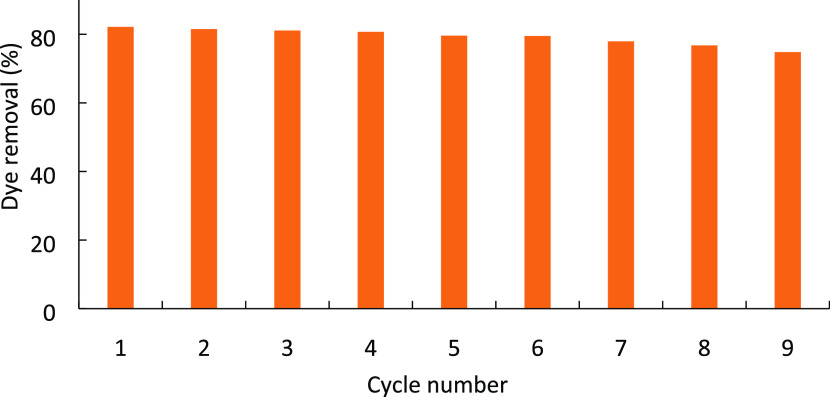
Reusability of the CeO_2_-NPs/GO/PAM hydrogel.

The capability to reuse a photocatalyst is critical for cost-effective industrial use. The reusability of the CeO_2_-NPs/GO/PAM hydrogel was examined over nine repeat (10 in total) cycles as shown in Figure 15. The hydrogel was dried after the first degradation. The hydrogel was recontacted with the MB solution under the determined optimized conditions. In the second use of the catalyst, the dye removal decreased to 85%. In the fifth repeat cycle, the photocatalyst was active with a >80% removal efficiency, which supports the reusability of hydrogels. In the ninth repeat cycle, 75% of the dye was decreased. Since the degradation efficiency was decreased to 70% in the 10th use of the hydrogels, the results of nine repetitions were given.

## 4 Conclusions

In this study, CeO_2_ nanoparticles and GO-doped PAM hydrogels were synthesized. XRD analysis clearly indicates that the addition of CeO_2_ nanoparticles was successfully incorporated into the PAM hydrogel, which is further supported by the FTIR analysis. The produced hydrogel was employed for the photodegradation of methylene blue dye from an aqueous solution. In the optimized conditions, 90% dye degradation efficiency was observed. Considering its high dye removal efficiency and favorable reusability, it is anticipated that this hydrogel may be used in practical wastewater treatment.
